# Lithotomy

**Published:** 1864-04

**Authors:** 


LITHOTOMY.

You published last year a paper by the London surgeon whom I just mentioned—Mr. James Lane, "On Lithotomy in the Female Bladder," in favor of the vesico-vaginal incision. Dr. Sims considers that the facility and invariable success with which a cut in the vesico-vaginal septum may now be closed suggest this as "the only justifiable operation for stone in the female bladder" He performed this operation first in 1850. It has since been repeatedly performed in America by Dr. Emmett, of the Women's Hospital of New York, and by Dr. Emmett, of Connecticut. The simplicity, safety, and unfailing success of the operation are spoken of in warm terms.

The application of this to the parallel method of *recto-vesical lithotomy in the male,* is a subject worthy of careful consideration. Recto-vesical lithotomy *in the adult* is a proceeding which was used long before the introduction of metallic sutures, and was followed with modifications by Mr. Lloyd, of St. Bartholomew's. Without these sutures it was liable to a serious objection—the occasional persistance of recto-vesical fistula. The silver-wire sutures, however, promise to obviate this inconvenience. Dr. Sims has mentioned to me a case in which Dr. Bauer, of New York, operated by this plan in 1859, Dr. Sims putting in the sutures. He says: "The patient was placed on the left side, and my speculum was introduced into the rectum, exposing the anterior wall of the rectum, just as it would the vagina in the female. A sound was passed into the bladder. The Doctor entered the blade of a bistoury in the triangular space bounded by the prostrate, the vesiculæ seminales, and the peritoneal reduplication. He passed the finger through this opening, felt the stone, and removed it with the forceps without the least trouble. The operation was done as quickly and as easily as it would have been in a female through the vaginal septum. After the removal of the stone, Dr. Bauer kindly asked me to close the wound with silver sutures, which I did, introducing some five or six wires with the same facility as in the vagina. There was no leakage of urine. The patient recovered without the least trouble of any sort. The wires were removed on the eighth day, and on the ninth day the patient rode in a carriage with Dr. Bauer a distance of four or five miles, to call on and report himself to our distinguished countryman, Dr. Mott. The facility and safety of executing recto-vesical lithotomy, (except in children for anatomical reasons,) and the success of closing at once the cut by the introduction of metalic sutures, ought to make this the operation in the male."—*London Lancet.*

